# Retrospective clinical study on the efficacy and complications of interventional embolization in the treatment of scalp arteriovenous fistula

**DOI:** 10.3389/fneur.2024.1461341

**Published:** 2024-10-30

**Authors:** Wenliang Han, Kexin Yang, Wei Gao, Xuejun Wu, Ran Huo, Lei Xu

**Affiliations:** ^1^Department of Burn and Plastic Surgery, Shandong Provincial Hospital Affiliated to Shandong First Medical University, Jinan, Shandong, China; ^2^Department of Vascular Surgery, Shandong Provincial Hospital Affiliated to Shandong First Medical University, Jinan, Shandong, China; ^3^Department of Rehabilitation Medicine, Shandong Provincial Hospital Affiliated to Shandong First Medical University, Jinan, Shandong, China

**Keywords:** scalp arteriovenous fistula, interventional embolization, clinical review, efficacy, safety

## Abstract

**Introduction:**

Scalp arteriovenous fistula (AVF) is a rare and intricate vascular anomaly characterized by a direct connection between an artery and a vein, without an intervening capillary system. This anomaly can induce significant local hemodynamic changes and is associated with various complications, such as pain, a pulsatile mass, increasing swelling, and venous hypertension skin ulcerations which may be non-healing. This study aimed to evaluate the efficacy and safety of interventional embolization treatments for scalp AVF at Shandong Provincial Hospital.

**Methods:**

This retrospective clinical analysis assessed 21 patients who underwent interventional embolization between 2018 and 2024. Patients included were those treated in the vascular surgery department at Shandong Provincial Hospital, who had comprehensive medical records and follow-up data. Treatment methods, outcomes, and complications were thoroughly analyzed through patient medical records.

**Results:**

Among the patients studied, direct puncture was the most prevalent treatment method, employed in 42.86% (9/21) of cases, followed by various combinations of arterial, venous, and direct approaches. Ethanol, used in 85.71% (18/21) of the cases, demonstrated its broad efficacy and application in clinical settings. Immediate imaging post-treatment confirmed a cure rate of 85.71% (18/21). The main postoperative complications included swelling, with some patients also experiencing nodules, scabbing, or hair loss.

**Conclusion:**

Interventional embolization has proven to be a safe and effective method for managing scalp AVF, significantly minimizing complications. Future research should focus on further optimizing these treatment methods to enhance efficacy and improve patient quality of life.

## 1 Introduction

Scalp Arteriovenous Fistula (AVF) is a rare vascular anomaly characterized by abnormal direct connections between arteries and veins, without an intervening capillary system. This condition often manifests as abnormal connections between the superficial scalp arteries and veins, involving any scalp blood vessels (1–3). These abnormal vascular structures can lead to local hemodynamic changes, producing pulsatile masses, and can cause severe clinical complications such as varying degrees of bleeding, pain, or skin ulcers (4). Scalp AVF, a rare and complex disease that can affect individuals of all ages, poses significant challenges in the fields of plastic surgery, neurosurgery, vascular surgery, interventional radiology, and interventional neuroradiology.

Currently, treatment options for scalp AVF include surgical resection, blood vessel ligation, vascular embolization, focal injection of sclerosing agents, and electrocoagulation thrombosis (5–9). These treatment modalities, either alone or in combination, yield different clinical outcomes. With advancements in medical technology, interventional embolization has emerged as a new and effective treatment option, offering advantages such as minimal invasiveness, high safety, and good efficacy (10–12).

Some patients with scalp arteriovenous fistulas (AVFs) have congenital spontaneous occurrence (generally developing from a red birthmark into a pulsatile mass), while others occur after trauma. These patients often have different degrees of bleeding as well as symptoms such as headache and tinnitus. Most of the reports in previous literature consist of individual case reports, but there is relatively little literature on systematic review studies regarding the effectiveness of injecting absolute ethanol for the treatment of scalp AVFs.

This study retrospectively analyzes the cases of scalp AVF treated with interventional embolization at Shandong Provincial Hospital’s Department of Vascular Surgery between 2018 and 2024. The aim is to provide a detailed discussion of the clinical practice of interventional embolization, evaluate its efficacy and complications, and offer comprehensive insights into the treatment of scalp AVF.

## 2 Methods

### 2.1 Patient population

This retrospective clinical analysis evaluates the efficacy and safety of interventional embolization for scalp AVF at Shandong Provincial Hospital’s Department of Vascular Surgery from 2018 to 2024. The inclusion criteria were patients treated at the hospital with complete medical records and follow-up data. Exclusion criteria included patients treated with non-interventional embolization methods or those with incomplete data. Patients with postoperative recurrent and residual arteriovenous fistulas were also included. These patients exhibited various signs and symptoms such as local erythema, scalp vasodilation, ulceration, and headaches. Written informed consent was obtained from each patient or their guardians to inform them of the benefits and risks of the surgery. The study was waived by the Ethics Committee due to the retrospective nature of the study, which did not require informed consent. The procedures for this study follow the Helsinki Declaration.

### 2.2 Data collection

Data were collected from the patients’ medical records, including gender, age, predisposing factors, onset time, lesion location, whether internal carotid artery branches were involved in blood supply, treatment approaches (e.g., direct puncture, arterial approach, venous approach), embolization materials used (e.g., detachable coils and non-detachable coils, absolute ethanol, glue, microspheres), immediate imaging results, and post-treatment complications.

### 2.3 Statistical analysis

After screening, 21 patients (18 males, 3 females; aged 7–45 years, despite being a congenital vascular malformation, patients may initially be asymptomatic and later develop symptoms. Our patient population has had a more than one year duration of the various symptoms) were included in the study. The specific data analysis is presented in the Results section and in Tables 1, 2.

**Table 1:** Patients with scalp arteriovenous malformations (AVMs): patient and AVMs characteristics.

Patient no./Gender/Age (y)	Cause	Duration of illness	Location of leision	Involvement of the internalcarotid arteryartery branches
1/M/45	No	10 years	RT	No
2/M/23	No	10 years	LO	No
3/M/45	Trau	2 months	LO	No
4/M/7	No	6 years	F	No
5/M/26	No	3 months	RT	No
6/F/16	No	10 days	P-O	No
7/M/38	Trau	7 years	LT	No
8/M/25	No	2 years	RF	No
9/M/3	IT	16 days	RFT	No
10/M/38	No	5 months	LT	No
11/M/12	No	18 days	F	No
12/F/33	No	10 months	F	No
13/M/18	No	17 years	LTP	Ophthalmic artery
14/F/22	No	3 years	RFT	No
15/F/39	No	2 years	T	No
16/M/16	No	10 years	LFT	No
17/M/19	No	16 years	RO	No
18/M/17	No	1 years	LF	No
19/M/45	Trau	5 months	LO	No
20/M/38	No	1 years	LT	No
21/M/19	No	16 years	RO	No

No., number; M, male; F, female; Trau, Cause Traumatism; IT, Iatrogenic Traumatism; RT, Location of leision Right temporal part; LO, Left occipital part; F, Frontal part; P-O, Parieto-occipital part; LT, Lefttemporal part; RF, Right frontal part; LTP, Left temporoparietal; RFT, Right frontotemporal; T, Temporal part; LFT, Left frontotemporal; RO, Right occipital part; LF, Left frontal part.

**Table 2:** Summary of interventional embolization with surgery in 21 patients with scalp AVMs.

Patient no./Gender/Age (y)	Approach	Controllable coils	Detachable coils	Alcohol	Glue	Microspheres	Immediate imaging results	Complications
1/M/45	DP	5	10	Yes	No	No	Cure	Swell
2/M/23	A, V, DP	2	8	Yes	Yes	No	Cure	Swell
3/M/45	A, V, DP	3	7	No	No	No	3	No
4/M/7	DP	2	3	Yes	No	No	Cure	Swell, gelosis
5/M/26	DP	0	4	Yes	No	No	Cure	Swell, gelosis
6/F/16	A, DP	0	0	Yes	Yes	No	Cure	swell, alopecia
7/M/38	A, DP	2	11	Yes	No	No	Cure	Swell
8/M/25	DP	0	5	Yes	No	No	Cure	Swell, gelosis
9/M/3	A	0	1	No	Yes	No	Cure	Scab
10/M/38	A, V	3	0	No	No	No	6	No
11/M/12	DP	0	0	Yes	Yes	No	Cure	Swell
12/F/33	DP	0	0	Yes	No	No	Cure	Swell, scab
13/M/18	V, DP	13	0	Yes	No	No	Cure	Swell, scab, alopecia
14/F/22	A, V, DP	1	2	Yes	Yes	No	Cure	Swell
15/F/39	A, DP	0	0	Yes	No	Yes	Improve	Swell
16/M/16	V, DP	5	35	Yes	No	No	Cure	Swell, gelosis
17/M/19	DP	6	0	Yes	No	No	Cure	Swell, alopecia
18/M/17	DP	2	0	Yes	No	No	Cure	Swell
19/M/45	A, DP	0	0	Yes	Yes	No	Cure	Swell
20/M/38	DP	3	16	Yes	Yes	No	Cure	Swell
21/M/19	A	0	0	Yes	No	No	Cure	Swell, scab, alopecia

AVMs, arteriovenous malformations. Approach Direct Puncture (DP), Arterial pathway (A), Venous pathway (V). Immediate imaging results Recurrence 3 months after surgery (3), Recurrence 6 months after surgery (6).

### 2.4 Treatment methods

Preoperative auxiliary examinations such as complete blood count, blood biochemical indices, coagulation function, and electrocardiogram were performed. Continuous monitoring of blood pressure, electrocardiogram, and blood oxygen saturation was conducted before and after treatment. All 21 embolization procedures were performed under general anesthesia with oral intubation. Serial digital subtraction angiography (DSA) was used to monitor the embolization process, providing detailed anatomical and hemodynamic information about the lesion. This allowed for timely evaluation of therapeutic effects and management of possible complications to achieve optimal outcomes and minimize risks.

Initially, patients underwent femoral artery puncture under anesthesia, with the catheter advanced to the carotid artery using radiographic image-guided technology. Contrast agent injection and arteriography provided detailed anatomical and hemodynamic information about the lesion, allowing for the determination of arterial and venous structures involved in the blood supply and drainage of the lesion. Based on the condition of the draining vessels, local puncture or continued access through the femoral vein was selected. For significant draining veins, 18G needles were used for direct puncture of the abnormal vascular mass and reflux veins, followed by micro catheter insertion through the needle. Peripheral non-detachable coils were first released, followed by detachable coils,. The coils are placed to partially obstruct and to slow AV shunting vascular flow to allow better ethanol intravascular contact, to denude the endothelial cells from the vascular wall (artery/vein) and precipitate their protoplasm and reduce the ethanol volumes required to achieve that goal. This then causes platelet aggregation on the denuded vascular wall by platelet accumulation peripherally to centrally to ultimately thrombose the vessel. This approach helped block abnormal blood flow, and improve the efficacy of absolute ethanol while minimizing its dosage. Absolute ethanol was injected after coil implantation. It should be noted that coil implantation does not damage vascular tissue. It causes and promotes intravascular thrombosis, not “damage.” Ethanol DOES intravascular damage by denuding the vascular wall of endothelial cells and precipitating their protoplasm, causing fractures of the vascular wall to the level of the internal elastic lamina, and the denuded/fractured wall that then has platelet aggregation that causes the thrombosis.

**Figure 1:**
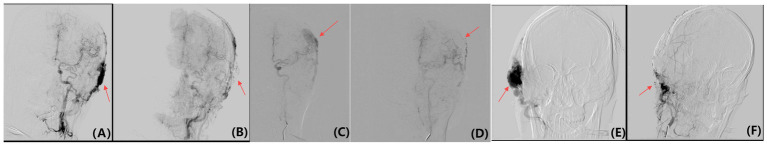
Pre-embolization DSAs and immediate post-embolization DSAs results of Yakes Type IIa AVM. (A) The head area shows obvious arteriovenous fistula, with a clearly visible abnormal blood vessel network, displaying vasodilation and circuitous characteristics, indicating a high flow of abnormal blood flow. This situation usually causes symptoms such as headache or skin ulcer and bleeding. (B) After embolization, the abnormal vascular network is significantly reduced. Vascular enhancement significantly diminishes, indicating that abnormal blood flow has been successfully blocked or reduced, and the vasodilation and circuitous characteristics have been relieved. (C) The head area shows obvious arteriovenous fistula, with a clearly visible abnormal blood vessel network, displaying vasodilation and circuitous characteristics. These abnormal blood vessels have remarkable enhancement, indicating a high flow of abnormal blood flow. (D) The abnormal vascular network is significantly reduced. Vascular enhancement significantly diminishes, indicating that abnormal blood flow has been successfully blocked or reduced. Vasodilation and circuitous characteristics have been relieved, showing that embolization materials effectively closed the arteriovenous fistula. (E) The left side of the head near the temporal area shows obvious abnormal arteriolar characteristics, indicating a high flow of arteriovenous shunt. The area indicated by the arrows shows a direct connection between the arterial and venous shunt, with some blood entering the Nidus and some draining from the Nidus through dilated veins. (F) DSAs was performed immediately after embolization.

For patients without significant bulky draining veins, direct local puncture was chosen, supplemented by temporary occlusion of blood flow, extrinsic manual compression will limit vascular flow (“velocity”), but it increases the intravascular pressure proximal to the compression in the area desiring to be embolized. Distal and downstream to the manual compression it does decrease intravascular pressure due to the proximal occlusion limiting inflow, until collaterals distally replace that flow volume to this area. Superselective imaging assessed whether normal tissue-supplying vessels were present. If present, embolization through the venous route or local puncture was performed to avoid damaging normal tissue. For lesions with independent blood supply, where all vessels supplied only the lesion (Nidus), arterial route injection of absolute ethanol was chosen. Careful angiographic evaluation ensured that only the diseased tissue was affected. Intraoperative imaging monitored the distribution and efficacy of the embolization agent, ensuring complete closure of the lesions while maintaining blood flow integrity to surrounding normal tissues. Once confirmed, treatment was concluded, with continuous observation post-operation to ensure other complications, the original abnormal arteriolar characteristics have mostly disappeared, showing significantly fewer high-flow blood vessels and improved arteriovenous shunt, with arterial blood supply and venous drainage expansion and distortion significantly improved, and the blood flow distribution returning to a normal pattern (Figure 1).

## 3 Results

Several incident factors have been noticed that have initiated the patients’ symptoms such as trauma (19.05%), however, in the majority of patients (80.95%) presenting with symptoms related to their AVFs no incident event was noted. This indicates that trauma is an important factor in the pathogenesis of acquired AVF (Table 1).

### 3.1 Onset time distribution

The onset of patients varied from days to years, indicating that scalp AVF can occur at any time without a specific morbidity peak (Table 1).

### 3.2 Lesion site analysis

The most common lesion site was the temporal region, accounting for 28.57% (6/21). The frontal, occipital, and multiple regions each accounted for 23.81% (5/21). This suggests that the richly vascularized collateral network in the scalp area can cause lesions to appear in multiple locations on the scalp (Table 1).

### 3.3 Involvement of internal carotid artery branches

We observed that in most cases of scalp AVF, branches of the internal carotid artery were not involved in the blood supply. Only one case (4.76%) involved the ophthalmic artery. Fully evaluating the blood flow path allows for more accurate treatment strategies and more effective closure of abnormal blood flow, reducing complications (Table 1).

### 3.4 Treatment pathway analysis

Direct puncture was the most commonly used method, accounting for 42.86% (9/21) of all cases. This method is preferred due to its ease of operation and high efficiency in directly targeting the lesion area. Direct puncture allows precise delivery of embolic material to the abnormal Nidus, effectively reducing abnormal blood flow. Additionally, the combination of arterial and direct puncture methods and the combination of arterial, venous, and direct puncture methods accounted for 19.05% (4/21) and 14.29% (3/21) of all treatments, respectively. These combined methods, involving multiple vessels, enhance therapeutic effects, particularly for complex or extensive AVF. Other approaches, including combined arterial and venous methods, simple arterial methods, and venous combined with direct puncture, accounted for 4.76% (1/21), 9.52% (2/21), and 9.52% (2/21), respectively. These data reflect adjustments in treatment strategies based on clinical cases and demonstrate the applicability and potential of different approaches in specific scenarios. Although direct puncture is a major treatment option, the combined use of arterial and venous approaches also shows clinical value in complex cases. Comprehensive embolization using different methods can more fully close abnormal blood flow, especially important for complex structures or cases where previous treatments failed. This multi-route strategy improves treatment comprehensiveness, providing multiple options for achieving optimal clinical outcomes and enhancing patient quality of life (Table 2).

### 3.5 Embolization material usage

Absolute ethanol was used in 85.71% (18/21) of patients, reflecting its widespread clinical use and effectiveness. Absolute ethanol is reliable for quickly and effectively closing abnormal blood vessels by destroying endothelial cells and promoting thrombosis, effectively controlling the condition. The novelty of this study lies in its evaluation of the efficacy of absolute ethanol in the treatment of AVF (arteriovenous fistula) based on a single-center retrospective analysis with a relatively large sample size. The results demonstrate that absolute ethanol is an effective treatment option, showing significant therapeutic outcomes. Detachable coils and non-detachable coils were also widely used, with a utilization rate of 71.5% (15/21). Coils physically block arterial or venous blood flow, providing long-term, stable embolization effects, particularly useful for complex vascular lesions. Glue embolization was used in 33.33% (7/21) of patients, demonstrating its advantages in precise embolization in certain cases. Glue materials rapidly solidify upon contact with blood, forming solid blocks suitable for precise vascular area occlusion. Microspheres were less commonly used, accounting for only 4.76% (1/21). Although effective for small vessel embolization, larger flow or diameter vessels may require more powerful materials to ensure efficacy (Table 2).

### 3.6 Analysis of real-time imaging results

To ensure accuracy and objectivity, international standard imaging results were used. Three senior doctors independently evaluated the images, each with extensive experience in scalp AVF analysis. They independently assessed post-treatment images for changes in blood flow, structural improvement, and potential anomalies. Their assessments were recorded separately and pooled. Consensus among the three radiologists was considered the final imaging result. In case of divided evaluations, they reviewed the images jointly and reached a consensus through discussion, consulting additional expertise if necessary. According to this standard, the majority of patients (85.71%) achieved a cured effect after treatment. One patient (4.76%) showed significant improvement in key clinical symptoms and quality of life but was not completely cured, necessitating continued follow-up. During postoperative follow-up, two patients relapsed at 3 and 6 months post-surgery, respectively. Despite embolization providing long-term effects for most patients, some may experience recanalization or incomplete thrombosis, requiring consideration of long-term follow-up and assessment in treatment planning (Table 2).

### 3.7 Complications

We conducted follow-ups at 1 month, 3 months, 6 months, and 1 year post-procedure. Postoperative complications primarily included swelling, with some patients experiencing lumps, scabbing, or hair loss. Only 9.52% (2/21) of patients had no obvious complications post-embolization, indicating that while interventional treatment effectively controls scalp AVF, it carries certain risks. Common issues such as postoperative swelling and lumps may result from local reactions and blood flow changes, while scabbing and hair loss may relate to skin damage in the operation area (Table 2).

## 4 Discussion

Scalp arteriovenous malformations (AVMs) are abnormal arteriovenous communications located within the subcutaneous fat layer of the scalp, forming a complex network of abnormal vessels and representing a rare and complex vascular disorder (13). There are two types of scalp AVFs: congenital and acquired. Congenital scalp AVF, also known as arteriovenous malformation, forms during early embryonic differentiation, with limited development leading to direct arteriovenous communication. This results in immature arteriovenous malformation with intertwined and dilated vessels, commonly located in the head and neck (14). Acquired scalp AVF often results from trauma or local piercing history (15, 16). In our study, eight congenital scalp AVM patients exhibited symptoms during adolescence, while the remaining nine showed symptoms in adulthood. Additionally, four cases (19%) developed secondary to scalp trauma.

Surgical resection is the classic treatment for scalp AVF (5), particularly indicated for bleeding prevention, cosmetic concerns, and accompanying tinnitus and headache (7, 17). However, due to the difficulty of complete surgical eradication and frequent recurrence or progression, surgical treatment alone has become less common (6, 18, 19). With advancements in interventional radiology and interventional neuroradiology, endovascular embolization plays an increasingly important role in AVF treatment (10–12). Vascular embolization reduces or eliminates AVF blood supply by introducing embolic materials into abnormal vessels to block blood flow, reducing rupture risk and improving symptoms (20). Conventional embolic materials include metallic coils, calibrated microspheres, and bioglue (21). Due to the complexity of scalp AVF, selecting appropriate access and embolization materials is crucial.

The scalp’s rich network of collateral vessels means that occluding major malformation nidus may not be sufficient, as blood may re-enter the diseased area through other routes. Therefore, treatment often involves venous embolization or blocking to directly address abnormal blood flow. In some cases, local direct puncture and precise embolization may be necessary, combining approaches to achieve optimal results (22–24). Half of the patients underwent embolization through two or more access routes. When it comes to the choice of embolic materials, liquid agents such as NBCA and Onyx polymerize and solidify quickly upon contact with blood, forming durable emboli that effectively occlude abnormal vessels. However, there remains a risk of recanalization over time (25). Particulate agents like polyvinyl alcohol (PVA) particles are capable of embolizing smaller vessels but may not completely block blood flow and carry the potential to migrate downstream with the blood flow (26). In recent years, the safety of absolute ethanol has been increasingly recognized. It causes direct damage to vascular endothelial cells and induces protein denaturation, leading to rapid and permanent vessel closure, making it an effective choice for treating arteriovenous malformations (AVMs) with minimal risk of recanalization (27–31). Dosage control is essential to prevent excessive embolization and tissue damage. High blood flow AVF may require combined embolization materials (e.g., coils) to physically reduce flow and enhance contact with embolic agents, preventing postoperative complications (32, 33).

### 4.1 Influence of approach and method

#### 4.1.1 Arterial approach

**Figure 2:**
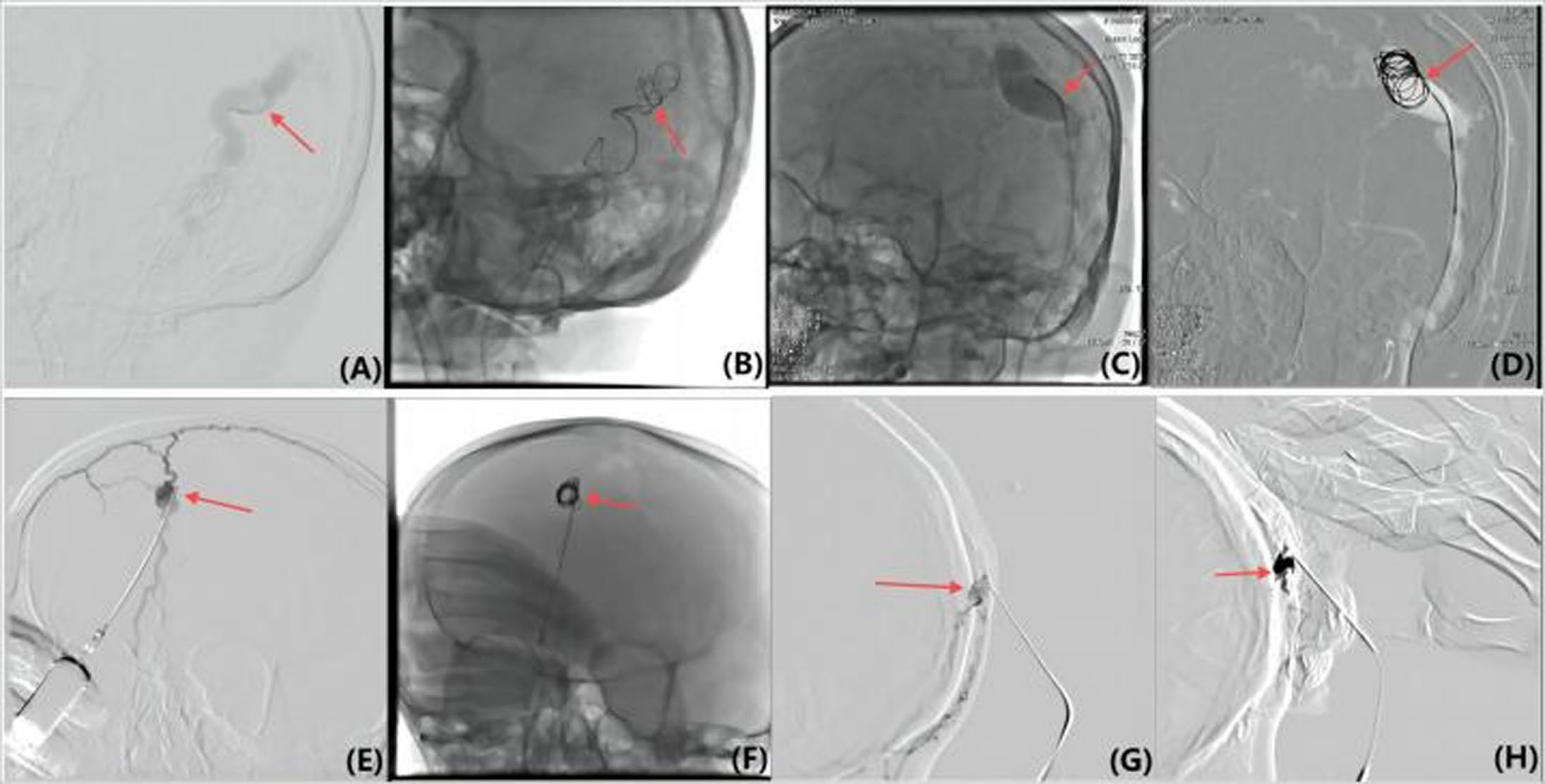
(A,B) Various methods were used to achieve flow control in the lesion, including arterial superselection into Nidus and coil insertion (C,D), reverse superselection through the external jugular vein and coil insertion (E,F), local lesion puncture and micro catheter implantation of the coil (G,H), and assisting finger compression to block the draining vein and reduce flow velocity.

Directly accessing the lesion core (Nidus) through the arterial route can be challenging due to arterial tortuosity and narrowing, resulting in a lower success rate and difficulty in achieving complete dense embolization (Figures 2A,B).

#### 4.1.2 Venous reverse approach

Higher success rate due to relatively flat veins, but requires spanning multiple vascular branches, necessitating high skill and experience (Figures 2C,D). The thin walls of veins require caution to prevent rupture and other complications.

#### 4.1.3 Direct puncture approach

Offers direct and convenient access but is challenging for finer venous drainage, with higher risk during catheter insertion and coil placement (Figures 2E,F). Consideration of vascular condition and risk–benefit balance is crucial (34).

#### 4.1.4 Local compression and suture

Effective for small draining veins, providing temporary flow restriction but not long-lasting and may involve high radiation exposure (Figures 2G,H). Efficacy and potential risks must be weighed.

### 4.2 Complications

#### 4.2.1 Facial edema

Common during recovery, related to the distribution of draining veins from AVF. Postoperative swelling is often more pronounced on the affected side, particularly around the eyes. Acute swelling typically lasts about a week, with complete resolution taking 4–6 weeks. Semi-recumbent positioning and medications like Seven Ye Zao glycosides, flavonoids, and traditional Chinese medicine can help reduce swelling. The amount of absolute ethanol used also correlates with swelling severity. For patients experiencing postoperative swelling, it generally subsides on its own. The specific recovery time varies depending on individual differences, but most patients show noticeable reduction in swelling within 2 weeks.

#### 4.2.2 Epidermal necrosis, scab, and scar

Closely related to factors like the degree of venous blockage, arterial reflux observed during local puncture angiography, the amount of absolute ethanol, and injection speed. Excessive ethanol can cause severe local reactions and extensive scarring. Complications occur from inadvertent non-target arterial embolization and occlusion of normal capillary beds supplying normal tissues. AVMs/AVFs supply NO tissues therefore occluding them causes no tissue injury issues. Despite not supplying tissues, extensive outflow vein occlusions can lead to venous injury/infarction, particularly in the skin/dermis. Four patients experienced post-procedural hair loss. While hair loss was not fully reversible in all cases, patients with unresolved localized hair loss managed it by changing hairstyles, undergoing hair transplants, or using wigs. Despite the hair loss, it did not significantly impact their quality of life. Three patients experienced scabbing post-procedure. The scabs required minimal intervention and resolved naturally as the wounds healed and the scabs fell off, allowing patients to return to normal life.

#### 4.2.3 Coil occupancy and exposure

Correct placement of coils is crucial to blocking blood flow and minimizing risks. Improper placement can cause coil migration, partial blockage, or recanalization, while exposed coils can lead to vascular injury, infection, and thrombosis.

Congenital scalp AVM and acquired AVF may become difficult to distinguish on imaging angiography as the disease progresses. Both conditions involve abnormal arteriovenous connections, but their clinical presentation and treatment strategies can differ. AVMs may exhibit a more complex Nidus, while AVFs typically have a direct arteriovenous connection. Local skin erythema, often developing into pulsatile masses with bleeding, is common. Venous hypertension caused by AVFs leads to vein expansion and appearance distortion, with symptoms like tinnitus, headaches, and scalp ulceration. Trauma, partial excision, arterial blockage, and endocrine changes can cause rapid progression.

Coils combined with absolute ethanol effectively treat scalp AVF, reducing blood flow. However, complications are related to ethanol amount, injection route, and speed. Accurate calculation and control are essential to prevent treatment failure and complications. Detailed angiography ensures precise ethanol delivery. Injection speed must be controlled to avoid tissue damage and maximize embolization efficacy.

## 5 Conclusion

This study demonstrates that interventional embolization is an effective and relatively safe method for treating scalp AVF. Despite short-term complication risks, correct embolization material and technique choices can significantly improve treatment success and safety. Future research should optimize these treatments to enhance efficacy and patient quality of life.

## Data Availability

The original contributions presented in the study are included in the article/supplementary material, further inquiries can be directed to the corresponding authors.
